# Acute low back pain is marked by variability: An internet-based pilot study

**DOI:** 10.1186/1471-2474-12-220

**Published:** 2011-10-05

**Authors:** Pradeep Suri, James Rainville, Garrett M Fitzmaurice, Jeffrey N Katz, Robert N Jamison, Julia Martha, Carol Hartigan, Janet Limke, Cristin Jouve, David J Hunter

**Affiliations:** 1 Division of PM&R, VA Boston Healthcare System, Boston, USA; 2 Spaulding Rehabilitation Hospital Network, 125 Nashua Street, Boston, USA; 3 New England Baptist Hospital, 125 Parker Hill Avenue, Boston, USA; 4 Department of Physical Medicine and Rehabilitation, Harvard Medical School, Boston, USA; 5 Department of Biostatistics, Harvard School of Public Health, Boston, USA; 6 Division of Rheumatology, Immunology and Allergy, Department of Medicine, Brigham and Women's Hospital, Harvard Medical School, Boston, USA; 7 Department of Orthopedic Surgery, Brigham and Women's Hospital, Harvard Medical School, Boston, USA; 8 Departments of Anesthesiology and Psychiatry, Brigham and Women's Hospital, Harvard Medical School, Boston, USA; 9 New England Research Institutes, 9 Galen Street, Watertown, USA; 10 Northern Clinical School, The University of Sydney, Sydney, Australia

## Abstract

**Background:**

Pain variability in acute LBP has received limited study. The objectives of this pilot study were to characterize fluctuations in pain during acute LBP, to determine whether self-reported 'flares' of pain represent discrete periods of increased pain intensity, and to examine whether the frequency of flares was associated with back-related disability outcomes.

**Methods:**

We conducted a cohort study of acute LBP patients utilizing frequent serial assessments and Internet-based data collection. Adults with acute LBP (lasting ≤3 months) completed questionnaires at the time of seeking care, and at both 3-day and 1-week intervals, for 6 weeks. Back pain was measured using a numerical pain rating scale (NPRS), and disability was measured using the Oswestry Disability Index (ODI). A pain flare was defined as 'a period of increased pain lasting at least 2 hours, when your pain intensity is distinctly worse than it has been recently'. We used mixed-effects linear regression to model longitudinal changes in pain intensity, and multivariate linear regression to model associations between flare frequency and disability outcomes.

**Results:**

42 of 47 participants (89%) reported pain flares, and the average number of discrete flare periods per patient was 3.5 over 6 weeks of follow-up. More than half of flares were less than 4 hours in duration, and about 75% of flares were less than one day in duration. A model with a quadratic trend for time best characterized improvements in pain. Pain decreased rapidly during the first 14 days after seeking care, and leveled off after about 28 days. Patients who reported a pain flare experienced an almost 3-point greater current NPRS than those not reporting a flare (mean difference [SD] 2.70 [0.11]; p < 0.0001). Higher flare frequency was independently associated with a higher final ODI score (*ß *[SE} 0.28 (0.08); p = 0.002).

**Conclusions:**

Acute LBP is characterized by variability. Patients with acute LBP report multiple distinct flares of pain, which correspond to discrete increases in pain intensity. A higher flare frequency is associated with worse disability outcomes.

## Background

The terms 'acute' and 'chronic' are commonly used in both research and clinical practice to characterize low back pain (LBP) and its prognosis. These terms as used in back pain research have been criticized, however, on the grounds that they overemphasize symptom duration and fail to capture important information about the experience of LBP and back-related disability[1-3]. Prospective studies have demonstrated that the experience of back pain is characterized by change and variation- through recurrences and remissions across pain episodes, and through flares of pain within pain episodes[3,4]. The fluctuating nature of back pain is often observed by clinicians caring for back pain patients, who are well familiar with individuals who return to clinic intermittently with flare-ups of pain during recent-onset, chronic, or recurrent LBP.

Flare-ups (or 'flares') of back pain have been defined as 'a period when back pain is markedly more severe than is usual for the patient'[1]. Flares of back pain in individuals with 'chronic' symptoms (>3 months) are associated with greater disability and work absenteeism, when adjusting for important factors, including pain intensity[5,6]. Pain *variability *therefore may be an important component of the pain experience that is related to disability, yet is distinct from pain intensity alone. Pain that is highly variable would be expected to have periods of relatively higher pain intensity (flares) and periods of relatively lower pain intensity; such fluctuations in pain intensity may increase the unpredictability of the pain experience, and may make pain more bothersome than pain that is stable and predictable. Nevertheless, pain variability in the acute LBP period (<3 months) has received limited study, with the majority of prior cohort studies not reevaluating patients until 1-3 months after the time of seeking care[7]. It is unknown whether discrete flares of pain from the course of acute LBP can even be discerned by patients in the context of the rapid improvement in pain intensity typical for acute LBP, which is distinct from the more stable baseline pain intensity expected in chronic LBP. A major obstacle to the study of flares and fluctuations of acute LBP is the logistical problem of how to go about documenting them. Even in the context of chronic LBP, flares of pain often last less than three days, and may recur frequently[6]. This creates a need for frequent assessments in order to 'capture' fluctuations of pain that may be recalled inaccurately- or not at all- when assessed retrospectively weeks to months later. However, frequent serial assessments using mailed questionnaires or telephone interviews has the potential to be both burdensome for participants to complete, and infeasible for researchers to administer.

To our knowledge, only a single study of acute LBP has employed frequent serial assessments permitting a characterization of pain fluctuations during the early course of acute LBP[8]. Sieben et al. followed 44 patients with acute LBP for the first 14 days after seeking care, in order to study changes in fear-avoidance beliefs during this period. Although this study demonstrated that flares of pain do exist in acute LBP, the authors did not characterize the frequency of flares, their occurrence over a more extended course after the first 14 days, or factors associated with flare occurrence. Furthermore, the use of written pain diaries to assess pain fluctuations in this earlier study posed a problem, since participants were not blinded to prior assessments. This had the potential risk of 'smoothing out' of variability due to the participant's ability to access and repeat the values used for earlier pain ratings. In addition, Sieben et al. did not examine associations between pain fluctuations and disability outcomes. This is important, because although much prior research has examined the prognostic utility of factors in predicting disability outcomes after acute LBP[9], no prior study has examined the role of acute pain variability on the transition to chronic back-related disability.

We proposed a pilot study to examine the feasibility of measuring pain variability and '*flares' *of pain during an acute LBP episode, using a cohort design with frequent serial assessments. We utilized Internet-based data collection specifically to improve the feasibility of the sampling scheme. The objectives of this pilot study were to characterize the fluctuations in pain intensity that occur during the course of an acute LBP episode, and to examine whether self-reported 'flares' of pain during acute LBP represented discrete periods of increased pain intensity which could be distinguished from periods when not in flare. Furthermore, we examined whether the frequency of flares was associated with back-related disability six weeks after seeking care for LBP, after accounting for other factors related to disability. In the course of this study, we evaluated the feasibility of maintaining high levels of subject compliance with frequent serial Internet-based assessments, during an acute pain episode.

## Methods

### Study Population

Study participants were recruited from the outpatient clinics of the Spine Center of New England Baptist Hospital. Consecutive patients age 18 and older with a diagnosis of acute low back pain were evaluated for participation. The majority of patients with LBP presenting to the Spine Center are direct patient self-referrals, although 20-25% of patients are referred by primary care physicians. Inclusion criteria required symptoms of new low back pain ≤ 3 months duration preceded by a substantial pain-free period lasting at least one month; literacy in English or Spanish; daily access to the Internet; basic computer literacy; and a valid email address accessible regularly for a period of 6 weeks. LBP was defined as either lumbar spinal pain or sacral spinal pain, as per recent consensus guidelines[10]. Exclusion criteria included radiating lower extremity pain experienced below the level of the knee; a predominant component of lower extremity pain greater than low back pain; motor weakness, asymmetric reflexes, or decreased sensation of the lower extremities associated with the current episode of LBP; symptoms of neurogenic intermittent claudication; clinical suspicion for 'red flag' conditions including infection, fracture, or neoplasm; recent low back trauma; pregnancy; severe active medical comorbidities or psychiatric illness. These exclusion criteria were chosen primarily to distinguish nonspecific back pain from two other broad categories of back pain: back pain potentially associated with radiculopathy or spinal stenosis, or back pain potentially associated with another specific spinal cause [11]; in addition, pregnant individuals were excluded due to the fact that LBP during pregnancy is likely to be affected by a variety of pregnancy-related factors and is therefore not nonspecific. Severe active medical comorbidities or psychiatric illness were criteria for exclusion because we expected these to make participation with data collection infeasible.

The Institutional Review Board of New England Baptist Hospital approved the conduct of this study and all study materials. Written informed consent was obtained for all individuals prior to study participation.

### Baseline Assessment- Demographics, Comorbidities, and Back Pain History

After informed consent was obtained, the examining physician used paper-based methods to record information on participant age, gender, race/ethnicity, medical and psychiatric comorbidity, employment status, and workers' compensation status. Race was categorized as 'Asian', 'Black', 'Hispanic', 'Native American or Alaskan Native', 'Pacific Islander', 'White', and 'Other'. Comorbidities were measured using the Self-Administered Comorbidity Questionnaire (SACQ). The SACQ is widely used in orthopedic research, and has previously demonstrated reliability and validity[12]. Employment status was categorized as part-time employment, full-time employment, student, retired, disabled, and unemployed.

Participants provided information on duration of current back pain symptoms and prior history of LBP episodes, lumbar disk herniation or sciatica, at the baseline evaluation. Current back pain intensity was measured by the numerical pain rating scale (NPRS) for back pain[13-15]. The NPRS is a 11-point scale in which patients rate their pain ranging from 0 (no pain) to 10 (worst imaginable pain). The NPRS is a valid and commonly used measure of back pain intensity that is simple for patients to comprehend and complete[13-15]. Back related disability was measured using the Oswestry Disability Index (ODI). The ODI is a condition-specific measure of disability which is used extensively in studies of low back pain, and has demonstrated validity and reliability in this context[16].

### Internet-Based Longitudinal Assessments- Flare Status and Back Pain Characteristics

Participants were sent a secure email link to an online survey tool at an email address they provided, on the day of the initial clinic evaluation. Survey Gizmo online survey software (Widgix, LLC, Boulder, CO) was used for all data collection after the in-clinic baseline assessment. Survey Gizmo is a web-based service that follows the Privacy Rule and Security Rule provisions of HIPAA (Health Insurance Portability and Accountability Act of 1996), and self-certifies adherence to HIPAA. HIPAA is a US federal law that establishes standards for the privacy and security of health information. Further information can be found at: http://www.surveygizmo.com/survey-blog/online-survey-hipaa-safe-harbor-certification. Participants were instructed to complete an initial online questionnaire as soon as possible after their initial clinic evaluation. Further online questionnaires were scheduled at both 3-day intervals and 7-day intervals after the date of completion of the first online questionnaire. The rationale for using 3-day intervals was to obtain more frequent measurements of back pain-related factors, consistent with our primary goal of characterizing short-term pain fluctuations. The 7-day interval was also included due to the fact that follow-up appointments are scheduled at one-week time intervals in standard clinical practice. The 3-day- and 7-day- questionnaires contained the same measures, with the exception of the ODI, which was included only at the 7-day intervals in order to minimize patient burden (see Additional File 1). Because we were aware of no prior information on the Internet-based serial assessment of individuals with acute LBP, we did not know whether it would be feasible to require individuals specific time windows within which to complete questionnaires. Participants were sent questionnaires on their scheduled date, with reminders after 1 day, and after 2 days. Responses were accepted at any time until the due date for completion of the next scheduled questionnaire.

Each online questionnaire assessed the presence or absence of a current pain flare. A flare of pain was defined as '*a period of increased pain lasting at least 2 HOURS, when your pain intensity is distinctly worse than it has been recently*'. This definition was adapted for acute LBP from the definition by Von Korff[1]. If reporting a current flare, participants described the duration of the current flare. Current back pain intensity was rated using the NPRS for back pain, and back-related disability was rated using the ODI. The Fear-Avoidance Beliefs Questionnaire[17] was used to assess the strength of participants' fear of back injury and relation to physical activity and work. The FABQ physical activity and FABQ work subscales have been validated for use in back pain patients [17]. Due to the length of the ODI and concerns regarding cumulative participant burden over the repeated assessments in the study, the ODI was administered only at weekly follow-up assessments. The FABQ was completed only at the first online questionnaire. The Internet-based data collection questionnaire items are provided in Additional File 1.

### Standard Medical Treatment of Acute LBP

All participants received standard medical care for nonspecific acute LBP as per practice guidelines[11]. Treatment included patient education, encouragement to avoid bed rest and normalize daily activities, use of oral analgesic medications when needed, and non-pharmacologic treatments as indicated[11].

### Statistical Analysis

Study participants who did not complete more than the first Internet questionnaire were considered 'non-compliant'. Non-compliant and compliant subjects were compared on demographic, medical/psychiatric, and back pain characteristics using the Student's t-test for quantitative variables and chi-square tests for categorical variables. The cohort of compliant subjects was characterized descriptively using means and standard deviations (SDs) for continuous variables, and frequencies and proportions for categorical variables. Due to the fact that few participants were from racial minority groups, race/ethnicity was categorized as white vs. non-white for all analyses.

Characterization of pain flares during the acute LBP episode was done by determining the prevalence of individuals who reported at least one flare, the number of discrete 'flares' reported by study participants, and the prevalence of flares of different durations. Next, we examined longitudinal changes in pain intensity as a function of time as days since seeking care, using mixed-effects linear regression models. Because longitudinal change in pain intensity during acute LBP has not been well-studied, and because existing knowledge suggests that much improvement occurs within the first 1-2 weeks[18,19], we first examined longitudinal trends in pain intensity using a linear spline model, including the predictor variables of time since seeking care (in days) and 'knots' (points at which spline slopes are permitted to change) at weekly intervals for six weeks. We then compared and contrasted the linear spline model with simple linear and quadratic models for the predictor variable of time since seeking care. We used the Akaike Information Criteria (AIC) as an indicator of model fit, and chose the most parsimonious model that demonstrated good fit. We then applied this model, while including the time-varying covariate of patient self-report of a current pain flare, to examine the longitudinal association of flare status with pain intensity. We computed *ß *coefficients and standard errors (SEs). Although change in back pain intensity was the focus of this pilot study, we also characterized longitudinal change in ODI scores using the same analytic methods described above.

Longitudinal analyses were followed by complementary multivariate regression analysis to model associations between flare frequency and the outcome of disability, while adjusting for other possible predictors of disability outcomes. Disability was measured as the final ODI score at study completion. Because some participants missed questionnaires towards the end of the study, the last value for ODI score was carried forward, provided that it was within 1 week of study completion. Flare frequency was defined as the number of flare periods reported by each participant, divided by the total number of questionnaires completed by the participant, expressed as a percentage. We first examined the bivariate association between flare frequency and the outcome of ODI using linear regression. Next, we used separate bivariate linear regression models to examine relationships between potential adjustment factors and the outcome of ODI score. Potential adjustment factors included participant age, gender, medical/psychiatric comorbidity, baseline pain intensity, baseline ODI score, duration of symptoms, work status, FABQ physical activity, and FABQ work. Recognizing our limited sample size, adjustment factors to be included in the final multivariate model were chosen by including only those predictor variables with the strongest bivariate associations with the outcome of ODI score. We included in the final multivariate model the predictor variable of flare frequency, and those adjustment factors that demonstrated p-values < 0.20 in bivariate analyses, up to a maximum of four variables (as limited by our sample size). Adequacy of the fitted model was assessed by scatter plots of studentized residuals. We also conducted a sensitivity analysis using the same analytic approach with an alternate definition of the flare outcome as the total number of reported flares during the six-week follow-up period. Last, analogous linear regression analyses were performed using the same candidate predictor variables, but the outcome of pain intensity at six weeks.

## Results

Eleven out of 77 consecutive potential study subjects declined participation or were missed by recruiting physicians over a 9-month period. Of the 66 participants who consented to participate in the study, six subjects did not respond to the initial Internet-based questionnaire, and 13 subjects did not complete any questionnaires after the initial one. The 47 subjects who completed the initial Internet-based questionnaire and at least one follow-up questionnaire were considered to be 'compliant' to use of the Internet, and comprised the cohort which was followed longitudinally during from the course of acute LBP. Compliant subjects were more likely to be female (57% vs. 22%; p = 0.02) than noncompliant subjects, but were otherwise similar with respect to other factors, including age, race, employment status, comorbidities, pain intensity, and disability (data not shown). The baseline characteristics of the cohort that was followed longitudinally are demonstrated in Table 1. Missed questionnaires were common during the six-week follow-up period, with the average subject completing 12.7 ± 5.1 questionnaires, out of a total of 20 scheduled questionnaires. Subjects who completed more questionnaires were more likely to be currently working in a full-time or part-time role than those who completed fewer questionnaires (72% vs. 40%; p = 0.04), but were otherwise similar with respect to all other factors including baseline and final pain and disability (data not shown).

**Table 1:** Characteristics of the Study Stample (n = 47)

	**Mean (S.D.) **or **N (%)**
Age (yrs.)	49.7 (13.4)
Gender (% Female)	27 (57%)
Race (% White)	45 (95.7%)
Self-administered Comorbidity Questionnaire* (0-45)	3.1 (3.6)
Duration of symptoms (days)	20.0 (17.2)
Prior low back pain history* (%)	39 (87%)
Prior lumbar disk herniation or sciatica*	17 (39%)
Current or past significant tobacco use* (%)	5 (11%)
Employment Status*	
Current part-time employment (%)	5 (12%)
Current full-time employment (%)	24 (57%)
Unemployed	2 (5%)
Retired	6 (14%)
Disabled	5 (12%)
Worker's compensation* (%)	4 (9%)
Numerical Pain Rating Scale (0-10)	6.0 (2.4)
Oswestry Disability Index* (0-100)	46 (23)
Fear Avoidance Beliefs Questionnaire, Physical Activity Subscale*(FABQ-PA)	13.1 (5.0)
Fear Avoidance Beliefs Questionnaire, Work Subscale* (FABQ-WORK)	9.4 (8.7)

*Variable with missing data

**Figure 1:**
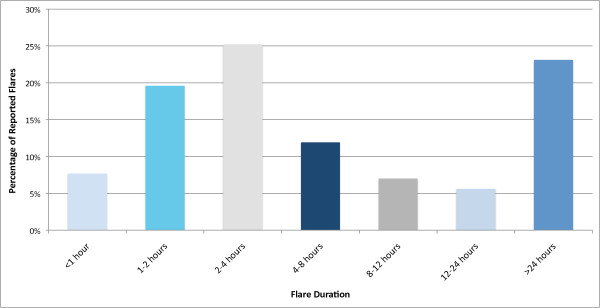
**Frequency of Flares of Different Duration**.

Pain flares were reported by 42 of 47 participants (89%) over six weeks of follow-up. 148 discrete pain flare periods were reported. Among those participants who reported pain flares, the average number of discrete flare periods was 3.5 (range 1-11) over the course of the study. Figure 1 depicts the frequency of flares of different duration. More than half of reported flares were less than 4 hours in duration, and about 75% of flares were less than one day in duration. Of the 23% of flares that were more than 24 hours in duration, the mean ± SD length of a flare was 8.1 ± 8.9 days.

**Figure 2:**
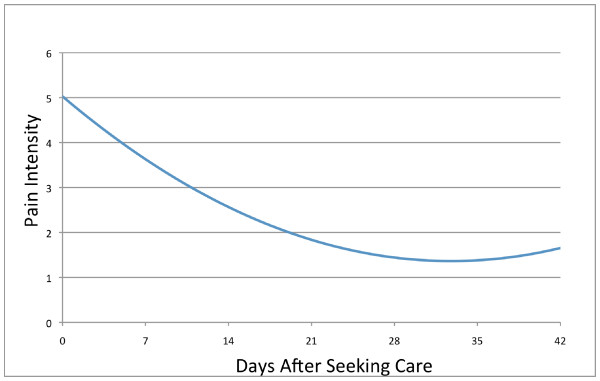
**The Course of Acute Low Back Pain Intensity**.

At six-week follow-up, mean ± SD back pain (1.4 ± 2.0) and disability scores (15 ± 16) demonstrated dramatic improvements over the severity of the clinical presentation at time of seeking care. Linear spline models using knots at 1-week intervals suggested that the majority of back pain improvement occurred over the 1^st ^week after seeking care, suggesting that a model assuming a linear time effect would not be appropriate. However, a model including a quadratic time trend (i.e., including terms for both time [days since seeking care] and time^2 ^since study entry) showed improved model fit (AIC = 2364) over a linear trend model (AIC = 2445), and over linear spline models with knots at one-week intervals (AIC = 2521), and with a single knot at one week (AIC = 2518). This quadratic trend over time indicated that pain intensity decreased rapidly during the first 14 days after seeking care (49% improvement from baseline), decreased at a lesser rate during the next 14 days (71% improvement from baseline), and leveled off after about 28 days; the predicted course of acute LBP intensity produced by this model is depicted in Figure 2. A model with a quadratic trend for time also produced slightly improved model fit over the linear model when reported flare periods were excluded from the analysis (data not shown). A model with a quadratic trend for time was therefore used in subsequent analyses to examine longitudinal relationships between patient-reported flares and pain intensity

Next, we used linear mixed-effects regression to model pain intensity over the six-weeks of follow-up as a function of time (days since seeking care), time^2^, and current flare status. Time (ß [SE] -0.091[0.014]; p < 0.0001) and time^2 ^(0.0013 [0.0003]; p < 0.0001) were highly significantly associated with longitudinal pain intensity. A self-report of a concurrent pain flare was also associated with a large marginal increase in current pain intensity (*ß *[SE] 2.70 [0.11]; p < 0.0001). That is, at any time during follow-up, patients who reported a flare experienced an approximate 3-point increase in pain intensity (on a 0-10 scale) as compared to those who did not experience a flare. This flare 'effect' was independent of the curvilinear trajectory of improvement imposed by the quadratic trend for time. Longitudinal changes in ODI score closely paralleled the course of pain intensity in acute LBP (Additional File 2).

In complementary multivariate linear regression analysis, we examined associations between flare frequency and baseline characteristics, and the outcome variable of ODI at 6 weeks (Table 2). Flare frequency was significantly associated with ODI score at 6 weeks in the bivariate analysis (*ß *[SE] 0.34 (0.08); p < 0.0001). This means that an increase of 10% in flare frequency was associated with a 3.4-point higher final ODI score at 6 weeks; similarly, an increase of 50% in flare frequency was associated with a 17-point higher ODI. In additional bivariate regression models, the factors of baseline ODI score and duration of symptoms also demonstrated associations with final ODI score, with p-values <0.10, and were therefore retained in the multivariate analysis. In multivariate analyses including the predictor variables of flare frequency, baseline ODI score and duration of symptoms, flare frequency (*ß *[SE} 0.27 (0.08); p = 0.003) remained significantly associated with ODI score, although the strength of the association was attenuated. The results of the multivariate analysis indicate that an increase of 50% in flare frequency was associated with a 14-point higher final ODI score, when adjusted for baseline ODI score and duration of symptoms. Duration of symptoms remained significantly associated with ODI score in the multivariate analysis, but baseline ODI score was not. This means that higher ODI scores at study entry were less strongly associated with ODI scores at study completion when adjusting for duration of symptoms and flare frequency. In sensitivity analyses using instead the outcome of total number of reported flares, results were not materially different. In analogous analyses using instead the outcome of pain intensity at six weeks, a similar relationship was seen between flare frequency and final pain intensity (Additional File 2).

**Table 2:** Predictors of Final ODI Score at 6 weeks*

	Bivariate Associations		Multivariate Associations	
**Predictor Variables**	***ß *[SE}**	***p*-value**	***ß *[SE}**	***p*-value**

Flare frequency^† ^(0-100%)	0.34 (0.08)	<0.0001	0.27 (0.08)	.003
Initial ODI Score (0-100)	0.28 (0.12)	0.02	0.18 (.11)	0.11
Duration of symptoms at presentation (days)	.002 (.001)	.09	0.002 (0.001)	0.05

* Only predictor variables included in the final multivariate model are presented

^† ^Flare frequency defined as the number of flare periods reported by each participant, divided by the total number of questionnaires completed by the participant, and expressed as a percentage.

## Discussion

**Figure 3:**
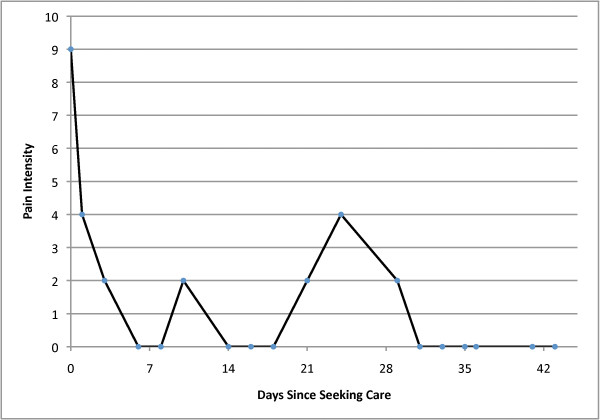
**Example #1 of Flares During the Course of Acute Low Back Pain**.

**Figure 4:**
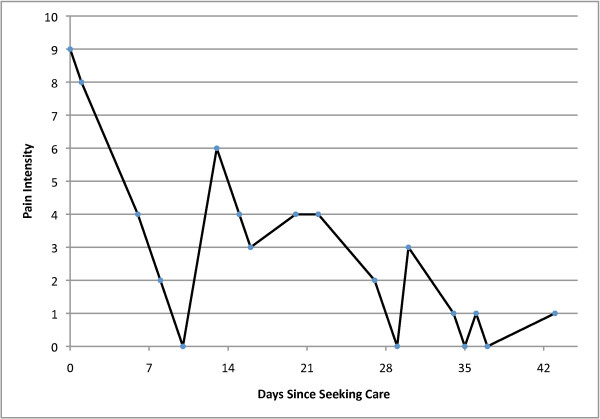
**Example #2 of Flares During the Course of Acute Low Back Pain**.

Multiple, discrete flares of increased pain were experienced by nearly all individuals recovering from acute LBP. Although the majority of reported flares lasted less than 24 hours, almost one quarter of flares lasted about one week. The time course of back pain improvement was curvilinear, with the majority of improvement occurring in the first two weeks after seeking care, and a leveling off of improvement after 4 weeks. However, despite the fact that pain levels were constantly changing during acute LBP, individuals were able to identify pain flares that corresponded to statistically significant increases in pain intensity as compared to other individuals not reporting flares, with a flare 'effect' of almost 3 points on the NPRS at any time during follow-up. This flare effect exceeds the threshold of 2 NPRS points commonly accepted as a minimal important change in NPRS[20]. The high degree of variability typical during acute LBP is difficult to accurately conceptualize using descriptions of flare frequency and magnitude of common pain intensity changes; Figures 3 and 4 present two examples of individual patient data that better convey the degree of variability seen during acute LBP.

Our study findings fit well into the context of prior cohort studies of acute LBP, which have shown dramatic improvements in pain at 1-2 weeks after seeking care[18,19]. Sieben et al. demonstrated a rather gradual course of improvement in pain intensity over 2 weeks after seeking care for many individuals, although fluctuations in pain were noted, and individuals with increasing levels of pain-related fear did not have decreases in pain[8]. Our study confirms that most patients have a rapid improvement in pain intensity, but not to complete resolution. Furthermore, flares of pain occur even after pain has largely improved, and for many individuals, multiple discrete episodes of transient pain worsening occur. Since early improvement is accompanied by the potential for brief returns to higher-intensity pain levels, individuals with recently improved pain intensity may be hesitant to advance function immediately after pain becomes more tolerable. A possible connection between flares and disability is supported by the results of our secondary, cross-sectional analysis, which found that individuals who had a higher flare frequency had greater disability at study end, irrespective of other factors including baseline disability or duration of symptoms.

A recent study of chronic LBP flares found that the presence of flares was associated with higher levels of disability even when adjusting for demographic factors and pain intensity[6]. Independent of other factors, individuals with flares were found to have high levels of maladaptive coping, which is an important predictor of long-term disability in LBP[9]. Although coping was not assessed in the current study, our finding of an association between higher flare frequency and disability raises the question of whether pain variability and maladaptive coping are related in the acute LBP period. Such a relationship could have a number of possible explanations. Flares- which occur when pain is variable- may be a pain experience that reinforces maladaptive coping or fear-avoidance. Indeed, it seems logical that pain variability would lead to less predictable pain, posing a greater problem for those with passive coping rather than adaptive coping skills. Alternatively, individuals with poor coping skills may be predisposed to experiencing greater pain variability for other reasons. Despite much research into factors that may predict the acute-to-chronic transition in LBP, no prior research has investigated pain variability as a prognostic factor. Future prognostic research should examine pain variability, as well as interrelationships with other known risk factors for chronicity, including maladaptive coping.

This pilot study demonstrates the feasibility of maintaining subject compliance with repeated data collection during an acute pain episode. Compliant subjects were more likely to be female and more likely to be working, but were otherwise not materially different from non-compliant subjects. However, missed questionnaires and late-completion questionnaires did occur, creating a potential for recall bias. Future studies may benefit from measures to improve compliance with questionnaire reminders utilizing modes of communication other than the Internet, such as phone calls or text message reminders. In addition, more sophisticated measures may be employed to limit recall bias, such as application of stringent, automated time constraints for questionnaire completion.

There are several limitations of this study that deserve mention. First, our use of frequent serial assessments and the fact that this was a pilot study required that this be a relatively small sample of patients. Second, some concerns apply regarding generalizability. Although these participants were recruited from a specialty spine clinic, some assurance of generalizability to primary care settings is provided by similarities between our study findings and those of the Sieben, which examined acute LBP with frequent serial assessments in a primary care setting. In addition, we included only nonspecific LBP, excluding cases of LBP with more specific causes such as radiculopathy, symptomatic lumbar spinal stenosis, trauma, infection, malignancy, fracture, and pregnancy; our findings may not be generalizable to acute LBP related to these causes. The use of the Internet raises a separate generalizability concern, but a growing literature suggests comparable reliability, internal validity, and external validity with use of the Internet as compared to conventional modes of data collection [21]. Although Internet-based data collection is not yet widely utilized in epidemiologic research, it offered several major advantages for the conduct of our study, given the frequent sampling employed, and the short time intervals between questionnaires. Internet-based questionnaires are returned more rapidly than postal questionnaires[22], which allowed us immediate notice of questionnaire completion, and the ability to send reminders for late questionnaires. Although telephone interviews might also have permitted this, Internet-based questionnaires take less time to complete[23], and minimize social acceptability bias. As previously mentioned, we wished to avoid the lack of blinding to earlier responses inherent in the use of written pain diaries. The use of electronic hand-held pain diaries may have provided additional benefits over our Internet-based approach, and future replication of our findings using these devices would be recommended. Third, as we mentioned, participants were allowed late completion of questionnaires more than 24 hours after notification was sent to complete the questionnaire. We made a pragmatic decision to allow this when planning the study, because we had no preliminary data to inform as to the feasibility of conducting frequent serial assessments of subjects in acute pain over the Internet. A prior electronic diary study reported that missed data points were not significantly associated with fluctuations in pain, mood, or stress[24], but were best explained by participants missing reminders to complete entries. This notion is supported by our finding that participants with fewer missed questionnaires were more likely to be working, and therefore to have access to email reminders. However, our study provides preliminary evidence of the feasibility of utilizing the Internet to closely follow patients with acute musculoskeletal pain. A final limitation of this study is that psychosocial factors including mood and coping, as well as objective functional measures that may have contributed to flares, were not assessed.

## Conclusions

Despite these limitations, this study demonstrates that the course of acute LBP is characterized by variability. Patients with acute LBP often have multiple distinct flares of pain in the six weeks after seeking care, and these flares correspond to discrete increases in pain intensity relative to periods not in flare. Future research should examine possible relationships between pain variability and long-term outcomes, as well as identify those demographic and psychosocial factors that are related to pain variability.

## Competing interests

The authors declare that they have no competing interests.

## Authors' contributions

PS was involved with study concept and design, acquisition of data, analysis of data, interpretation of data, and drafting of the manuscript. DJH was involved with study concept and design, interpretation of data, and manuscript preparation. JR was involved with study concept and acquisition of data. GMF was involved with study design and manuscript preparation. JNK was involved with interpretation of data. RJ was involved with study concept and design, and interpretation of data. JM, CH, JL, and CJ were involved with acquisition of data. All authors were involved with critical revision of the manuscript for important intellectual content and approved the final version of the manuscript.

## Pre-publication history

The pre-publication history for this paper can be accessed here:

http://www.biomedcentral.com/1471-2474/12/220/prepub

## Supplementary Material

Additional file 1 **Questionnaires**. Questionnaire schedule and examples of content. Click here for file

Additional file 2 **Additional Results**. Longitudinal changes in Oswestry Disability Index (ODI) score, predictors of final back pain intensity, and graphical comparison of ODI scores and pain intensity over 6 weeks. Click here for file
