# Supplementary material for: Acute low back pain is marked by variability: An internet-based pilot study
Source: BMC Musculoskelet Disord. 2011 Oct 5;12:220. doi: 10.1186/1471-2474-12-220 (PMC3198993; doi:10.1186/1471-2474-12-220)
Supplement: Additional file 1 — Questionnaires. Questionnaire schedule and examples of content. [file 1471-2474-12-220-S1.DOC]

**Supplemental Table 1- Schedule of Questionnaire Administration**

| **Day** | | **0** | | **3** | | **6** | | **7** | | **9** | | **12** | | **14** | | **15** | | **18** | | **21** | | **24** | | **27** | | **28** | | **30** | | **33** | | **35** | | **36** | | **39** | | **42** |
| --- | --- | --- | --- | --- | --- | --- | --- | --- | --- | --- | --- | --- | --- | --- | --- | --- | --- | --- | --- | --- | --- | --- | --- | --- | --- | --- | --- | --- | --- | --- | --- | --- | --- | --- | --- | --- | --- | --- |
| **Baseline Assessment**  (paper-based**)** | x | |  | |  | |  | |  | |  | |  | |  | |  | |  | |  | |  | |  | |  | |  | |  | |  | |  | |  | |
| **Short-form Questionnaire** (Internet-based) |  | | x | | x | |  | | x | | x | |  | | x | | x | |  | | x | | x | |  | | x | | x | |  | | x | | x | |  | |
| **Long-form Questionnaire^*^** (Internet-based) | x | |  | |  | | x | |  | |  | | x | |  | |  | | x | |  | |  | | x | |  | |  | | x | |  | |  | | x | |
| *Long-form questionnaire was identical to short-form questionnaire, except for inclusion of the Oswestry Disability Index | | | | | | | | | | | | | | | | | | | | | | | | | | | | | | | | | | | | | | |

**Supplemental Content 1- Presentation of Internet-based Data Collection Items**

The following information simulates the format of the Internet-based items for data collection that were presented to study participants. Please note that it does not duplicate the exact questionnaire.

A flare of low back pain is a period of increased pain **lasting at least 2 HOURS**, when your pain intensity is distinctly worse than it has been recently.

- **Are you currently having a flare of pain?**

|  | Yes |
| --- | --- |
|  | No |

- Please rate your pain by selecting the one number that tells how much pain you have **right now**.

| No Pain |  |  |  |  |  |  |  |  |  |  |  | Pain as bad as you can imagine |
| --- | --- | --- | --- | --- | --- | --- | --- | --- | --- | --- | --- | --- |
|  | 0 | 1 | 2 | 3 | 4 | 5 | 6 | 7 | 8 | 9 | 10 |  |

**🡪[IF FLARE]**

- - How long has this current flare of pain lasted?

|  | <1 hour |
| --- | --- |
|  | 1-2 hours |
|  | 2-4 hours |
|  | 4-8 hours |
|  | 8-12 hours |
|  | 12-24 hours |
|  | >24 hours |

- - Have you had more than one flare in the past 24 hours?

|  | Yes |
| --- | --- |
|  | No |

- - For approximately how many days has your current flare of pain lasted?
    (select from the drop-down menu)

[selection options 2-49)

- Pain intensity

|  | 0 - I have no pain at the moment. |
| --- | --- |
|  | 1 - The pain is very mild at the moment. |
|  | 2 - The pain is moderate at the moment. |
|  | 3 - The pain is fairly severe at the moment. |
|  | 4 - The pain is very severe at the moment. |
|  | 5 - The pain is the worst imaginable at the moment. |

- Personal care today (washing, dressing, etc.)

|  | 0 - I can look after myself normally without causing extra pain. |
| --- | --- |
|  | 1 - I can look after myself normally but it is very painful. |
|  | 2 - It is painful to look after myself and I am slow and careful. |
|  | 3 - I need some help but manage most of my personal care. |
|  | 4 - I need help every day in most aspects of self care. |
|  | 5 - I do not get dressed, wash with difficulty, and stay in bed. |

- Lifting

|  | 0 - I can lift heavy weights without extra pain. |
| --- | --- |
|  | 1 - I can lift heavy weights but it gives extra pain. |
|  | 2 - Pain prevents me from lifting heavy weights off the floor but I can manage if they are conveniently positioned, e.g. on a table. |
|  | 3 - Pain prevents me from lifting heavy weights but I can manage light to medium weights if they are conveniently positioned. |
|  | 4 - I can lift only very light weights. |
|  | 5 - I cannot lift or carry anything at all. |

- Walking

|  | 0 - Pain does not prevent me from walking any distance. |
| --- | --- |
|  | 1 - Pain prevents me from walking more than 1 mile. |
|  | 2 - Pain prevents me from walking more than 1/2 of a mile. |
|  | 3 - Pain prevents me from walking more than 100 yards. |
|  | 4 - I can only walk using a stick, crutches, cane or walker. |
|  | 5 - I am in bed most of the time and have to crawl to the toilet. |

- Sitting

|  | 0 - I can sit in any chair as long as I like. |
| --- | --- |
|  | 1 - I can sit in my favorite chair as long as I like. |
|  | 2 - Pain prevents me from sitting for more than 1 hour. |
|  | 3 - Pain prevents me from sitting for more than 1/2 hour. |
|  | 4 - Pain prevents me from sitting for more than 10 minutes. |
|  | 5 - Pain prevents me from sitting at all. |

- Standing

|  | 0 - I can stand as long as I want without extra pain. |
| --- | --- |
|  | 1 - I can stand as long as I want but it gives me extra pain. |
|  | 2 - Pain prevents me from standing for more than 1 hour. |
|  | 3 - Pain prevents me from standing for more than 1/2 hour. |
|  | 4 - Pain prevents me from standing for more than 10 minutes. |
|  | 5 - Pain prevents me from standing at all. |

- Sleeping

|  | 0 - My sleep is never disturbed by pain. |
| --- | --- |
|  | 1 - My sleep is occasionally disturbed by pain. |
|  | 2 - Because of pain I have less than 6 hours sleep. |
|  | 3 - Because of pain I have less than 4 hours sleep. |
|  | 4 - Because of pain I have less than 2 hours sleep. |
|  | 5 - Pain prevents me from sleeping at all. |

- Sex life (if applicable)

|  | 0 - My sex life is normal and causes no extra pain. |
| --- | --- |
|  | 1 - My sex life is normal but causes some extra pain. |
|  | 2 - My sex life is nearly normal but is very painful. |
|  | 3 - My sex life is severely restricted by pain. |
|  | 4 - My sex life is nearly absent because of pain. |
|  | 5 - Pain prevents any sex life at all. |

- Social life

|  | 0 - My social life is normal and causes me no extra pain. |
| --- | --- |
|  | 1 - My social life is normal but increases the degree of pain. |
|  | 2 - Pain has no significant effect on my social life apart from limiting my more energetic interests, e.g. sport, etc. |
|  | 3 - Pain has restricted my social life, and I do not go out as often. |
|  | 4 - Pain has restricted social life to my home. |
|  | 5 - I have no social life because of pain. |

- Travelling

|  | 0 - I can travel anywhere without pain. |
| --- | --- |
|  | 1 - I can travel anywhere but it gives extra pain. |
|  | 2 - Pain is bad but I manage journeys over two hours. |
|  | 3 - Pain restricts me to journeys of less than one hour. |
|  | 4 - Pain restricts me to short necessary journeys under 30 minutes. |
|  | 5 - Pain prevents me from travelling except to receive treatment. |

- My pain is caused by physical activity.

|  | 0 | 1 | 2 | Unsure 3 | 4 | 5 | 6 |  |
| --- | --- | --- | --- | --- | --- | --- | --- | --- |
| Completely Disagree |  |  |  |  |  |  |  | Completely Agree |

- Physical activity makes my pain worse.

|  | 0 | 1 | 2 | Unsure 3 | 4 | 5 | 6 |  |
| --- | --- | --- | --- | --- | --- | --- | --- | --- |
| Completely Disagree |  |  |  |  |  |  |  | Completely Agree |

- Physical activity might harm my back.

|  | 0 | 1 | 2 | Unsure 3 | 4 | 5 | 6 |  |
| --- | --- | --- | --- | --- | --- | --- | --- | --- |
| Completely Disagree |  |  |  |  |  |  |  | Completely Agree |

- I should not do physical activities which (might) make my pain worse.

|  | 0 | 1 | 2 | Unsure 3 | 4 | 5 | 6 |  |
| --- | --- | --- | --- | --- | --- | --- | --- | --- |
| Completely Disagree |  |  |  |  |  |  |  | Completely Agree |

- I cannot do physical activities which (might) make my pain worse.

|  | 0 | 1 | 2 | Unsure 3 | 4 | 5 | 6 |  |
| --- | --- | --- | --- | --- | --- | --- | --- | --- |
| Completely Disagree |  |  |  |  |  |  |  | Completely Agree |

- My pain was caused by my work or by an accident at work.

|  | 0 | 1 | 2 | Unsure 3 | 4 | 5 | 6 |  |
| --- | --- | --- | --- | --- | --- | --- | --- | --- |
| Completely Disagree |  |  |  |  |  |  |  | Completely Agree |

- My work aggravated my pain.

|  | 0 | 1 | 2 | Unsure 3 | 4 | 5 | 6 |  |
| --- | --- | --- | --- | --- | --- | --- | --- | --- |
| Completely Disagree |  |  |  |  |  |  |  | Completely Agree |

- I have a claim for compensation for my pain.

|  | 0 | 1 | 2 | Unsure 3 | 4 | 5 | 6 |  |
| --- | --- | --- | --- | --- | --- | --- | --- | --- |
| Completely Disagree |  |  |  |  |  |  |  | Completely Agree |

- My work is too heavy for me.

|  | 0 | 1 | 2 | Unsure 3 | 4 | 5 | 6 |  |
| --- | --- | --- | --- | --- | --- | --- | --- | --- |
| Completely Disagree |  |  |  |  |  |  |  | Completely Agree |

- My work makes or would make my pain worse.

|  | 0 | 1 | 2 | Unsure 3 | 4 | 5 | 6 |  |
| --- | --- | --- | --- | --- | --- | --- | --- | --- |
| Completely Disagree |  |  |  |  |  |  |  | Completely Agree |

- My work might harm my back.

|  | 0 | 1 | 2 | Unsure 3 | 4 | 5 | 6 |  |
| --- | --- | --- | --- | --- | --- | --- | --- | --- |
| Completely Disagree |  |  |  |  |  |  |  | Completely Agree |

- I should not do my normal work with my present pain.

|  | 0 | 1 | 2 | Unsure 3 | 4 | 5 | 6 |  |
| --- | --- | --- | --- | --- | --- | --- | --- | --- |
| Completely Disagree |  |  |  |  |  |  |  | Completely Agree |

- I cannot do my normal work with my present pain.

|  | 0 | 1 | 2 | Unsure 3 | 4 | 5 | 6 |  |
| --- | --- | --- | --- | --- | --- | --- | --- | --- |
| Completely Disagree |  |  |  |  |  |  |  | Completely Agree |

- I cannot do my normal work till my pain is treated.

|  | 0 | 1 | 2 | Unsure 3 | 4 | 5 | 6 |  |
| --- | --- | --- | --- | --- | --- | --- | --- | --- |
| Completely Disagree |  |  |  |  |  |  |  | Completely Agree |

- I do not think that I will be back to my normal work within 3 months.

|  | 0 | 1 | 2 | Unsure 3 | 4 | 5 | 6 |  |
| --- | --- | --- | --- | --- | --- | --- | --- | --- |
| Completely Disagree |  |  |  |  |  |  |  | Completely Agree |

- I do not think that I will ever be able to go back to that work.

|  | 0 | 1 | 2 | Unsure 3 | 4 | 5 | 6 |  |
| --- | --- | --- | --- | --- | --- | --- | --- | --- |
| Completely Disagree |  |  |  |  |  |  |  | Completely Agree |
