# Supplementary material for: Acute low back pain is marked by variability: An internet-based pilot study
Source: BMC Musculoskelet Disord. 2011 Oct 5;12:220. doi: 10.1186/1471-2474-12-220 (PMC3198993; doi:10.1186/1471-2474-12-220)

**Supplemental Content 2- Longitudinal Change in ODI Score**

Longitudinal change in ODI scores was best characterized by a model including a quadratic time trend, with improved fit over a linear trend model and linear spline models (data not shown). The predicted course of disability as measured by the ODI is presented in Supplemental Figure 1. This demonstrates that the course of disability very closely parallels the course of pain intensity in acute LBP, albeit with a gentler initial slope of improvement for disability as compared to pain intensity. When using linear mixed-effects regression to model disability over the six-weeks of follow-up as a function of time, time^2^, and current flare status, time (*ß* [SE] -0.85[0.16]; p <0.0001), time^2^ (0.011 [0.003]; p=0.0006), and having a self-reported concurrent pain flare (10.91 [1.54]; p <0.0001) were highly significantly associated with ODI score. That is, at any time during follow-up, patients who reported a flare had an approximate 11-point increase ODI score as compared to those who did not have a flare.

**Supplemental Table 2. Predictors of Final Back Pain Intensity at 6 weeks^*^**

|  | **Univariate Associations** | | **Multivariate Associations** | |
| --- | --- | --- | --- | --- |
| **Predictor Variables** | ***ß* [SE}** | ***p*-value** | ***ß* [SE}** | ***p*-value** |
| Flare frequency^†^  (0-100%) | 0.04 (0.01) | 0.0003 | 0.04 (0.01) | .0004 |
| Initial Back Pain Intensity  (0-10) | 0.17 (0.12) | 0.15 | -0.04 (0.11) | 0.74 |
| Fear-Avoidance Beliefs Questionnaire (days) | 0.08 (.05) | 0.16 | 0.06 (0.05) | 0.21 |

* Only predictor variables included in the final multivariate model are presented

^†^ Flare frequency defined as the number of flare periods reported by each participant, divided by the total number of questionnaires completed by the participant, and expressed as a percentage.

'


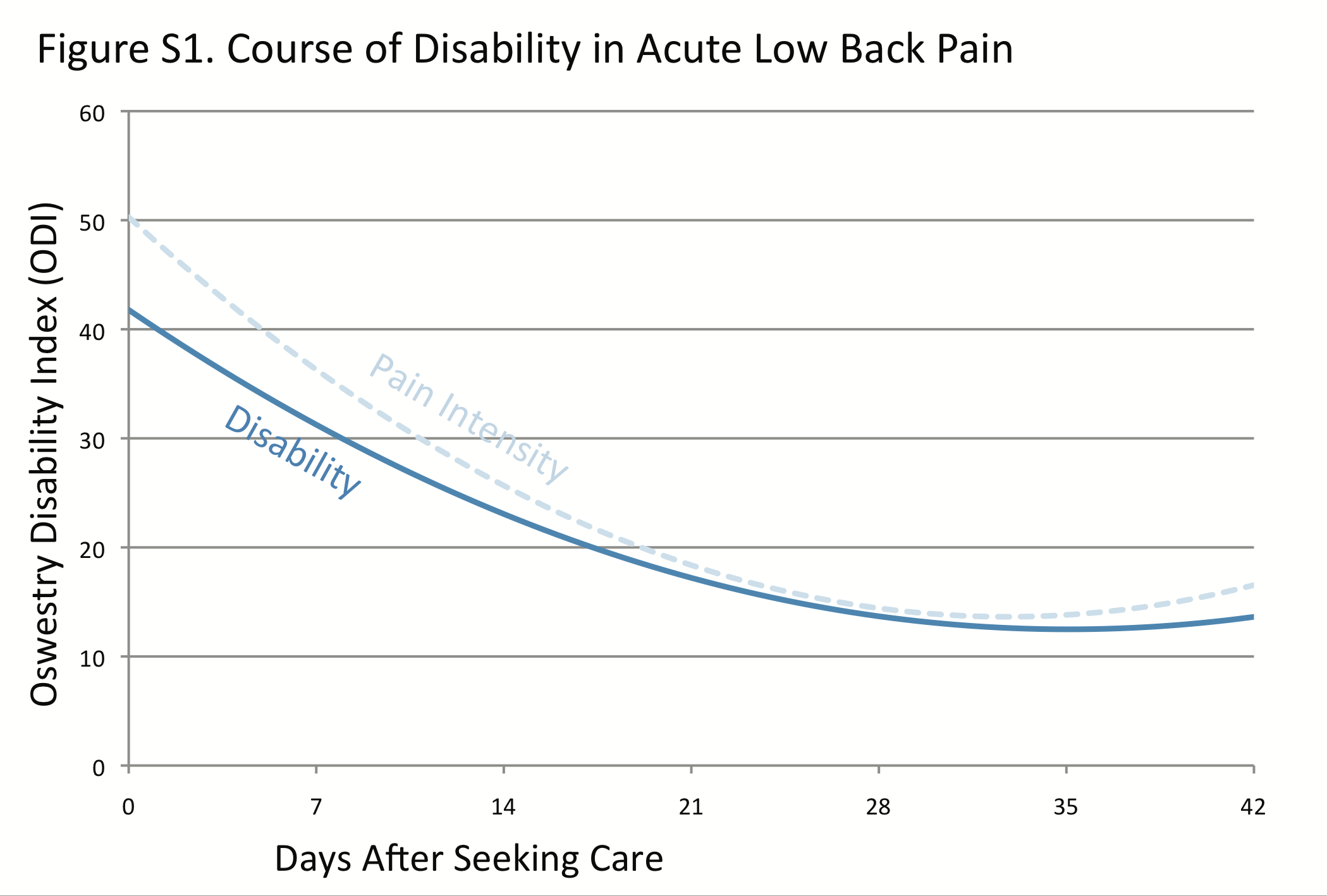

Supplement: Additional file 2 — Additional Results. Longitudinal changes in Oswestry Disability Index (ODI) score, predictors of final back pain intensity, and graphical comparison of ODI scores and pain intensity over 6 weeks. [file 1471-2474-12-220-S2.DOC]
